# Ten-year association between change in speech-in-noise recognition and falls due to balance problems: a longitudinal cohort study

**DOI:** 10.1186/s12889-024-18187-5

**Published:** 2024-03-07

**Authors:** Lotte A. Jansen, Marieke F. van Wier, Freek P. J. Vernimmen, Thadé Goderie, Raymond van de Berg, Ulrike Lemke, Birgit I. Lissenberg-Witte, Sophia E. Kramer

**Affiliations:** 1 Department of Otolaryngology – Head and Neck Surgery, Section Ear and Hearing, Amsterdam UMC Location Vrije Universiteit Amsterdam, De Boelelaan 1117, Amsterdam, the Netherlands; 2 Amsterdam Public Health Research Institute, Quality of Care, Amsterdam, the Netherlands; 3 Department of Epidemiology and Data Science, Amsterdam UMC Location Vrije Universiteit Amsterdam, Meibergdreef 9, Amsterdam, the Netherlands; 4 Department of Otorhinolaryngology and Head and Neck Surgery, Division of Vestibular Disorders, Maastricht University Medical Centre, P. Debyelaan 25, Maastricht, The Netherlands; 5 Research & Development, Sonova AG, Staefa, Switzerland

**Keywords:** Incident falls, Recurrent falls, Dizziness, Hearing ability, Longitudinal, Hearing aids

## Abstract

**Background:**

This study examined the relationship between speech-in-noise recognition and incident/recurrent falls due to balance problems ten years later (RQ-1); 10-year change in speech-in-noise recognition and falls (RQ-2a), as well as the role of dizziness in this relationship (RQ-2b). The association between hearing aid use and falls was also examined (RQ-3).

**Methods:**

Data was collected from the Netherlands Longitudinal Study on Hearing between 2006 and December 2022. Participants completed an online survey and digits-in-noise test every five years. For this study, data was divided into two 10-year follow-up time intervals: T0 (baseline) to T2 (10-year follow-up), and T1 (5-years) to T3 (15-years). For all RQs, participants aged ≥ 40 years at baseline, without congenital hearing loss, and non-CI users were eligible (*n* = 592). Additionally, for RQ-3 participants with a speech reception threshold in noise (SRTn) ≥ -5.5 dB signal-to-noise ratio were included (*n* = 422). Analyses used survey variables on hearing, dizziness, falls due to balance problems, chronic health conditions, and psychosocial health. Logistic regressions using General Estimating Equations were conducted to assess all RQs.

**Results:**

Among individuals with obesity, those with poor baseline SRTn had a higher odds of incident falls ten years later (odds ratio (OR):14.7, 95% confidence interval (CI) [2.12, 103]). A 10-year worsening of SRTn was significantly associated with a higher odds of recurrent (OR: 2.20, 95% CI [1.03, 4.71]) but not incident falls. No interaction was found between dizziness and change in SRTn. Hearing aid use (no use/ < 2 years use vs. ≥ 2 years) was not significantly associated with incident nor recurrent falls. Although there was a significant interaction with sex for this association, the effect of hearing aid use on incident/recurrent falls was not statistically significant among males nor females.

**Conclusions:**

A longitudinal association between the deterioration in SRTn and recurrent falls due to balance problems after 10 years was confirmed in this study. This result stresses the importance of identifying declines in hearing earlier and justifies including hearing ability assessments within fall risk prevention programs. Mixed results of hearing aid use on fall risk warrant further investigation into the temporality of this association and possible differences between men and women.

**Supplementary Information:**

The online version contains supplementary material available at 10.1186/s12889-024-18187-5.

## Introduction

Aging affects both balance [1] and hearing ability [2], and problems in both often coexist as age increases. Hearing [3] and balance issues [4] can lead to increased risk for falls, which are detrimental causes of injury and disability among older adults [5]. In 2020, one in three individuals above the age of 65 experienced one or more falls in the Netherlands [6]. The prevalence of falls has been shown to increase in middle-age, particularly for women where the prevalence increased from 8.7% among women aged 40–44 years to 29.9% aged 60–69 years across four population-based cohort studies [7]. The number of emergency visits in the Netherlands due to fall-related injuries among the elderly has also been increasing over time [8]. Falls can cause serious physical injuries, such as fractures, cerebral or visceral hemorrhages, and soft tissue injuries [5], as well as psychological consequences such as depression and reduced everyday functional level and quality of life [9]. Alongside individual consequences, the costs of fall-related injuries have a large impact for society, with total emergency medical costs being 1.1 billion euros in the Netherlands in 2020 [6]. Considering the personal and societal consequences of falls, preventive actions are imperative.

A wide number of risk factors for falls have been identified, including older age, female gender, high body mass index (BMI) [9], certain chronic health conditions, history of falling, visual impairment [5], dizziness [10], depression [9], alcohol consumption, social isolation, and loneliness [11]. Furthermore, hearing impairment has also been shown to be associated with postural balance problems and falls [12–15]. Possible explanations are reduced cognitive capacity for balance [16–18] and spatial awareness [19]. In one meta-analysis of four cross-sectional studies that assessed hearing loss using audiometry, hearing loss was associated with 1.72 higher odds of falling [20]. However, two longitudinal studies in this meta-analysis showed no significant difference in the odds of falls between individuals with and without hearing impairment, likely due to how hearing impairment was defined (inclusion of those with mild impairment). Additionally, it was previously found that dizziness might be a risk factor for falls [10, 15]. Dizziness complaints may also co-exist with hearing impairment [21]. One cross-sectional study of 2,750 patients with dizziness showed that vestibular dysfunction and non-vestibular-related dizziness were associated with a higher odds of falls compared to patients without dizziness complaints [22]. However, in those with dizziness complaints, no significant association between the degree of hearing impairment and odds of falls was found. Stam et al. [15] found that those with hearing impairment had a higher odds of “dizziness causing falling”, but did not examine dizziness and falls as separate factors, which is important as dizziness does not always cause falls and vice-versa. Current evidence for a causal relation between hearing loss and falls is mixed and the role of dizziness in this relationship is unclear. Longitudinal research examining whether a deterioration in hearing over time combined with vestibular symptoms increases the likelihood of falling is needed. It should be noted that, according to van de Berg et al. [23], vestibular symptoms include e.g., dizziness and unsteadiness (balance issues) and can also arise from disorders beyond the vestibular system (e.g. cardiovascular disorders). In vestibular medicine, dizziness can be defined as the sensation of disturbed or impaired spatial orientation without a false or distorted sense of motion, while ‘unsteadiness’ (balance issues) is the feeling of being unstable while seated, standing, or walking without a particular directional preference [24] (pp7,9). In laymen’s language, ‘dizziness’ and ‘unsteadiness’ may not be used in the clinical sense. In the Dutch language in particular, ‘dizziness’ can refer to a number of vestibular symptoms including dizziness defined as above as well as (clinically distinct) vertigo. Some individuals may also use the term dizziness while actually referring to unsteadiness and vice-versa. However, the majority of Dutch individuals distinguish the term ‘balance problems’ from dizziness, with balance problems referring to problems in keeping postural stability when standing or moving. Nevertheless, research on fall risk in the Netherlands benefits from taking both dizziness and balance issues into account in order to best capture individuals at risk for falls.

Hearing impairment could have a large impact on falls due to its high prevalence among older adults. The Global Burden of Disease estimates of 2019 show that approximately 25% of those above 60 years of age suffer from moderate to severe hearing loss (≥ 35 dB hearing loss in the better ear) globally [25]. Hearing impairment accelerates after the age of 50 years [26], with the prevalence for those aged 80–85 years being 52% and 90 + years being 91% in the Netherlands [27]. Those with severe and profound hearing impairment have higher mortality ratios, with death by trauma as one of the main causes of mortality compared to those with better hearing [28].

While major risk factors for falls are already targeted in fall-prevention programs, aural rehabilitation is one avenue that has yet to be explored. A meta-analysis of 283 randomized controlled trials (RCTs) found that combined exercise and vision assessment and treatment resulted in a significant reduction in fall-related injuries [29]. With this in mind, identification of hearing impairment may also be considered when assessing fall-risk. Additionally, little is known on whether ensuing aural rehabilitation with hearing aids (HAs) mitigates the risk of falls. Three reviews found equivocal evidence of an effect of hearing aid use on balance [12, 30, 31]. Other studies reported mixed results when assessing fall risk specifically, showing that hearing aid use (vs. no use) either reduced fall diagnosis/risk [32–34], increased it [35, 36], or had no statistically significant effect [37].

As hearing impairment tends to accelerate after the age of 50 years [26] and the incidence of nonfatal fall-related injuries has also been shown to accelerate around this age [7], it is important to assess the extent to which a *deterioration* in hearing may contribute to fall risk and related injuries, and whether hearing-aids could mitigate this risk. Due to the negative impact of falls on individuals and society, and the high prevalence of hearing impairment, examining the longitudinal association between the two could make a positive contribution to improving fall prevention programs and ultimately improve quality of life among adults at risk of falls. To address existing gaps in the literature, the present study will assess the following Research Questions (RQs):

- RQ-1: What is the association between baseline hearing ability and falls due to balance problems 10 years later among adults aged 40 years and older at baseline?
- RQ-2: (a) What is the association between 10-year change in hearing ability and falls at 10 years due to balance problems? (b) Does this association differ among adults with and without dizziness complaints?
- RQ-3: Is there a cross-sectional association between hearing aid use and falls among those with hearing impairment?

## Materials and methods

### Study design and setting

For this longitudinal study, quantitative data collected from the Netherlands Longitudinal Study on Hearing (NL-SH) between 2006 and December 2022 was utilized. The NL-SH is an online prospective cohort study that examines the association between hearing ability and (psychosocial) health, healthcare utilization, lifestyle, and participation in society. The study was started in 2006 and is currently ongoing. Adults between the ages of 18 and 70 years at time of enrollment, with and without hearing impairment, are eligible for participation. Participants are requested to complete an online survey and hearing is tested every five years (baseline (T0), 5-year (T1), 10-year (T2), and 15-year measurement rounds (T3)), or until voluntary disenrollment. In the current study the T0 measurement round serves as baseline for the 10-year follow-up at T2, and T1 as the baseline for the 10-year follow-up at T3, so two main 10-year time intervals were chosen: (i) T0-T2 and (ii) T1-T3. More details regarding the NL-SH have been described by van Wier et al. [38] The NL-SH study protocol was approved by the institutional review board (Medical Ethics Committee) of Amsterdam UMC, location VUmc in Amsterdam, the Netherlands (METC number 2006/83; NL12015.029.06).

### Measures

#### Dependent variables

**Fig. 1:**
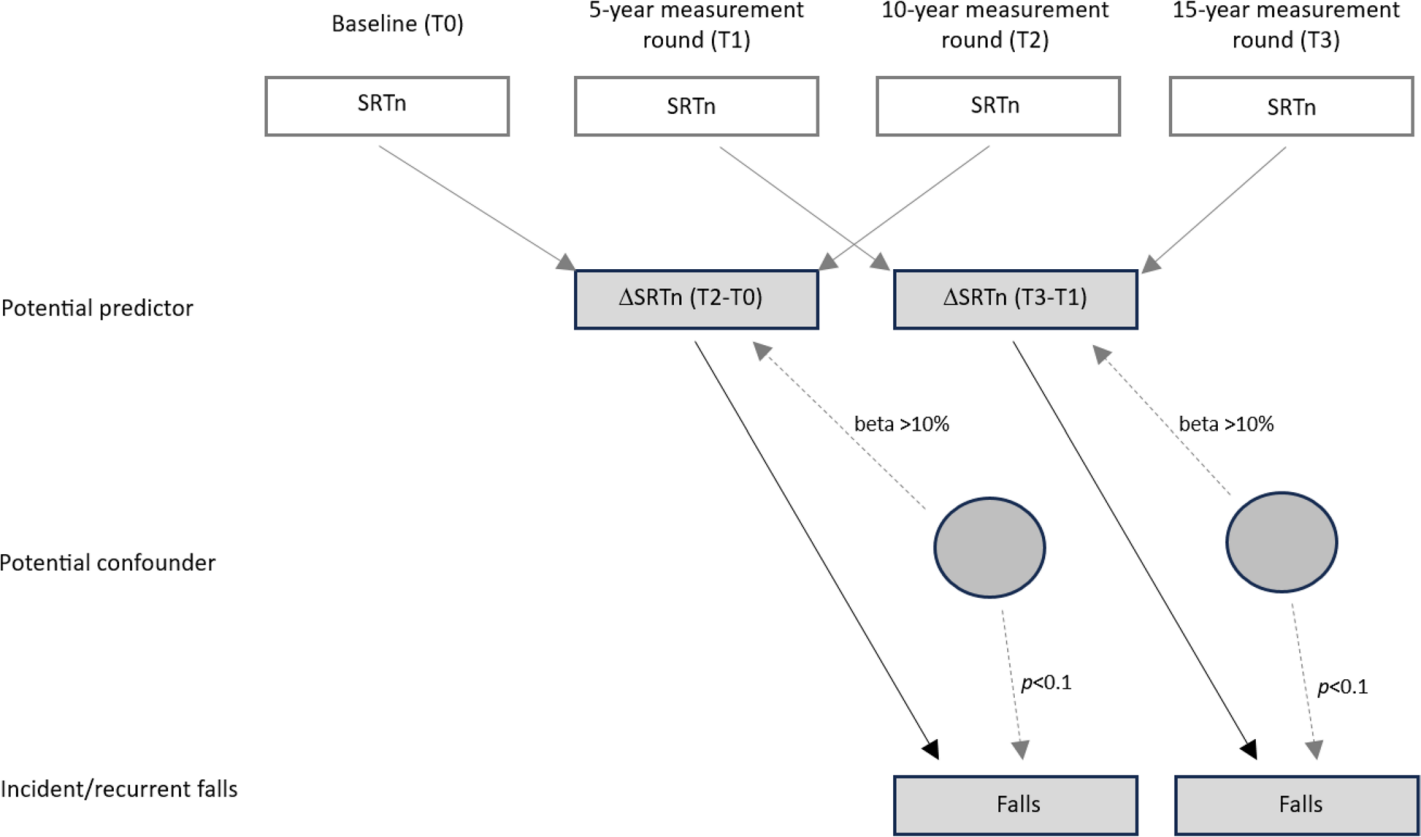
Illustration of NL-SH time points and the data utilized at each time point for RQ-2a, 2b. *Note*. All potential confounders were measured at T2 or T3, except for baseline SRTn, age, and sex, which were measured at T0 or T1. Fall data at T2 and T3 were utilized for analyses. The arrows from the four SRTn’s pointing at the two ΔSRTn’s show how the ΔSRTn’s were created. The remaining arrows depict the associations between the independent, dependent, and potential confounding variables. This figure is adapted from van Leeuwen et al. [54]. *RQ* research question, *NL-SH* Netherlands Longitudinal Study on Hearing, *ΔSRTn* change in speech reception threshold in noise

Figure 1 shows the relationships between all the main measures used in the study and time point(s) or measurement rounds from which they were taken and used for analyses. To measure falls, participants were asked the survey question, “How many times have you fallen due to balance problems during the past year?”, with answer options 0, 1, or ≥ 2 times at T2 (10-year measurement round) and an open field option (ranged from 0 to 365) at T3 (15-year measurement round). To examine the odds of experiencing *incident* falls (single event), these answer options were dichotomized into 0 (0 falls) and 1 (1 fall). To examine the odds of experiencing *recurrent*falls (multiple falls in the same year), the original answer options were dichotomized into 0 (0 or 1 falls) and 1 (≥ 2 falls). Recurrent falls may have greater clinical relevance than incident falls because recurrent fallers experience greater morbidity than those who are not recurrent fallers [39]. Both dependent variables were tested in separate regression models.

#### Independent variables

Speech reception threshold-in-noise (SRTn) was measured by the National Hearing Test, which is an online adaptive speech-in-noise test that measures the ability to understand 23 digit-triplets against a background of noise. Participants were requested to remove their hearing aids prior to beginning the test and recommended to use binaural headphones (though speakers were permitted). The average signal-to-noise ratio (SNR) of the last 20 triplets (which corresponds to 50% intelligibility) is used to calculate a participant’s SRTn score. SRTn correlates strongly with pure tone average (PTA) results, where *r* = 0.73 for PTA_0.5,1,2 kHz_ and *r* = 0.77 for PTA_0.5,1,2,4 kHz_. The known measurement error of the National Hearing Test within approximately 1 dB SNR [40].

When assessing the association between baseline SRTn and falls at 10 years (RQ-1), the independent variable used was baseline SRTn (T0 and/or T1). Because it was not linearly associated with falls, baseline SRTn values were categorized into quartiles: -10.6 to -7.4 dB SNR (quartile 1), -7.2 to -6.2 (2), -6.0 to -4.2 (3), and -4.0 to 1.9 dB SNR (4) of approximate size. Baseline SRTn was also assessed as a possible confounder when assessing the association between change in SRTn and falls after 10 years (RQ-2). For this RQ, the 10-year changes in SRTn (ΔSRTn) for each interval were calculated by subtracting the SRTn at baseline (T0) from the 10-year measurement round (T2) and SRTn at the 5-year round (T1) from the 15-year round (T3). These values were also divided into quartiles of approximately equal size: -8.8 to -1.0 dB SNR (quartile 1), -1.0 to 0.4 (2), 0.4 to 2.2 (3), and 2.2 to 8.6 dB SNR (4). Positive ΔSRTn scores indicate worsening of hearing over time.

When assessing the relationship between hearing aid use and falls at 10 years (RQ-3), survey questions “Do you use a hearing aid(s)?” (“yes” or “no”) and “How long have you used hearing aid(s)?” were used to create a new dichotomous variable, hearing aid use. This variable had categories 0 (no hearing aid use or hearing aid use of < 2 years) and 1 (≥ 2 years hearing aid use), and was measured at the time of reported falls (T2 or T3). A two-year cutoff was chosen to allow for sufficient time to measure a potential effect of hearing aid use on falls during the past year. Had we chosen a one-year period of hearing aid use, participants would have started using their hearing aid during the same year that self-reported falls were measured. In that case, falls could have occurred prior to wearing the hearing aid or during its adjustment period, which can take a few months. By measuring hearing aid use for at least two years, we can ensure that participants had a hearing aid adjustment period and reported falls after this adjustment period.

#### Confounders and effect modifiers

As fall-risk is multifactorial, the following variables were examined for confounding or effect modifying effects: *age* (years); *sex* (“male” or “female”); *living situation* (“alone” or “with others”); *level of education* (“low” or “mid-level” or “high”) [38]; *baseline SRTn (T0 or T1)*; *hearing aid use* (“none or < 2 years” or “ ≥ 2 years”); *depression levels* measured by the Four-Dimensional Symptom Questionnaire (4DSQ) [41] (“moderate to severe” or “not present to mild”); *loneliness levels* measured by the De Jong Gierveld Scale [42] (“moderate to severe” or “not present to mild”); *dizziness complaints* (“yes” or “no”); *history of dizziness with falls* (“yes” or “no”); presence of the following chronic health conditions (“present” or “not present”): *severe heart disease, hypertension*, *diabetes, chronic back pain*, *osteoarthritis of the knees and hips*, *rheumatoid arthritis*, *other chronic arthritis*, *nervous system disorders other than Parkinson’s*, *malignant condition or cancer, injury due to accident*; *near- or farsightedness* (“yes” or “no”); *obesity* (“body mass index (BMI) < 30″ or” ≥ 30″); *mobility issues* (“no issues”, “mild-moderate issues”) [43]; *difficulty performing usual activities* (“no issues”, “mild-moderate issues”) [43]; and *alcohol consumption* (“ ≤ 2 glasses” or “ > 2 glasses per drinking occasion”). Chronic health conditions were analyzed as separate factors in statistical models. All variables except for *baseline SRTn (T0 or T1)*, *age*, and *sex*, were measured at T2 or T3. For RQ-1 and 2, *hearing aid use* (“yes” or “no”) was included. For RQ-3, *SRTn at the moment of self-reported falls (T2 or T3)* was also measured. These variables were assessed due to their documented associations with hearing impairment [15, 44, 45] and fall risk [9, 11, 20, 39, 45–47].

Medication use, particularly polypharmacy and use of a variety of types of medications are associated with fall risk [48–53]. Up until 2021, participants could list up to 10 medications they used during the past four weeks, but these questions were omitted in the T3 questionnaire and not all participants answered these questions during previous measurement rounds, resulting in incomplete data. For sensitivity analyses, available data on medication use was categorized into different variables by whether they were known to increase fall risk, decrease risk, mixed results (both increase and decrease according to different studies), or were not related to fall risk [48–53]. Dichotomous variables for medication use at all (0 vs. ≥ 1 medications) and polypharmacy (1–3 vs. 4–10 medications) were also created (see SM Tables 1 and 2).

### Sample population

For all RQs, all 3024 participants of the NL-SH enrolled up to December 2022 were assessed for eligibility. Participants were eligible if they were aged 40 years or older at baseline (T0) or at 5-years (T1), completed the National Hearing Test at one or both of the intervals, and completed the fall-related survey questions at T2 and/or T3. Participants younger than 40 years of age at baseline (T0 or T1) were excluded to allow for a minimum age of 50 years at time of falls. This is because both hearing loss [26] and the incidence of fall-related injuries [7] accelerates around this age. Due to their hearing loss being profound and a low likelihood of detecting a further decline in hearing ability over time, participants were excluded if they had self-reported congenital hearing loss or used a cochlear implant. Moreover, participants with congenital hearing loss at birth or at an early age may allow for a certain level of adjustment to balance problems. By adulthood, congenital hearing loss may then have less effect on fall risk. Additionally, for RQ-1 (association between baseline SRTn and falls after 10 years) and 2 (association between ΔSRTn and falls), participants were excluded if they took the National Hearing Test while wearing a hearing aid as this may lead to unreliable results. Due to the inability of the hearing test to measure a SRTn of > 4 dB SNR, participants with a SRTn of ≥  + 2 dB SNR at baseline (T0 or T1) were excluded to allow for a 10-year SRTn change of at least 2 dB. If participants had an improbable ΔSRTn over 10 years (defined as ± 3 times the interquartile range (IQR) of ΔSRTn, equaling 9.0 dB SNR, they were excluded as well as this likely represents measurement error. For RQ-3 (association between hearing aid use and falls), participants with missing data on hearing aid use or non-users with good hearing ability (SRTn < -5.5 dB SNR) [55] were excluded. In the Supplementary Material (SM), Figs. 1 and 2 show how participants were excluded to obtain the final samples used for analyses.

### Statistical analyses

Descriptive statistics for independent, dependent, and sociodemographic variables were generated. To assess the associations between baseline SRTn as well as 10-year ΔSRTn and falls (RQ-1 and 2a, respectively), longitudinal logistic regressions were performed using Generalized Estimating Equations (GEE) with an exchangeable working correlation structure, which takes repeated measurements into account and assumes equal correlations between each pair of measurements. Variables with a minimum of ten observations were included in analyses. Crude analyses were performed to test for effect modification and confounding. First, we tested for effect modification by looking at whether the interactions between the independent variable and potential effect moderator were statistically significant (*p* < 0.1) in the crude regression model. If significant, regression results were reported per stratum of the effect moderator. Subsequently, we tested for confounding by checking whether (i) the confounder was significantly associated with the dependent variable and with the independent variable (*p* < 0.1) and if (ii) a ≥ 10% change in the beta coefficient of the independent variable was present after adding the confounder to the crude model [56]. After identifying confounders, adjusted analyses were reported. To assess whether the presence of dizziness significantly affected the association between 10-year ΔSRTn and falls (RQ-2b), we analyzed the interaction between dizziness and ΔSRTn on falls. Odds ratios and 95% confidence intervals were reported.

To examine the cross-sectional association between hearing aid use and falls (RQ-3), a logistic regression was performed using GEE and the same statistical steps were taken to assess the presence of effect modification and confounding. Odds ratios (OR) and 95% confidence intervals (CI) were reported.

Sensitivity analyses were also performed using univariable GEE analyses to assess whether the categorical medication variables described in “Confounders and Effect Modifiers” were significantly associated with ΔSRTn and with fall risk separately, as these are conditions that warrant evaluation for confounding effects. In case of significant associations, feasibility to impute missing medication data was evaluated to enable analyses with medication as a possible confounder using the full sample. All analyses were conducted using IBM SPSS Statistics 28.

## Results

### Sample characteristics

Table 1 shows descriptive data for the study participants for each research question.

**Table 1:** Sample characteristics for RQs 1 (association baseline SRTn and falls), 2a (association ΔSRTn and falls after 10y), 2b (role of dizziness) and 3 (association hearing aid use and falls)

		RQ-1, 2a, 2b		RQ-3	
		T0-T2 (*N* = 496)	T1-T3 (*N* = 284)	T2 (*N* = 363)	T3 (*N* = 147)
Age (years), M ± SD (range)		53.0 ± 6.6 (40–71)	55.2 ± 7.8 (40–69)	53.6 ± 6.7 (40–69)	57.6 ± 7.5 (40–71)
Sex, *n* (%)	Female	280 (56.6)	164 (58.0)	220 (60.6)	90 (61.2)
Living situation, *n* (%)	Alone	120 (24.2)	72 (25.4)	78 (21.5)	36 (24.5)
Level of education, *n* (%)	Low or medium	191 (38.6)	106 (37.3)	150 (41.3)	54 (36.7)
Baseline SRTn, M ± SD		-5.57 ± 2.61	-5.40 ± 2.66	-2.19 ± 3.08	-1.27 ± 3.51
Baseline SRTn, *n* (%)	-10.6 to -7.4 dB SNR	135 (27.2)	69 (24.3)	-	-
	-7.2 to -6.2 dB SNR	116 (23.4)	76 (26.8)	-	-
	-6.0 to -4.2 dB SNR	124 (25.0)	66 (23.2)	-	-
	-4.0 to 1.9 dB SNR	121 (24.4)	73 (25.7)	-	-
ΔSRTn, M ± SD		0.62 (2.63)	0.58 (2.61)	-	-
ΔSRTn, *n* (%)	-8.8 to -1.0 dB SNR	133 (26.8)	86 (30.3)	-	-
	-1.0 to 0.4 dB SNR	122 (24.6)	67 (23.6)	-	-
	0.4 to 2.2 dB SNR	125 (25.2)	71 (25.0)	-	-
	2.2 to 8.6 dB SNR	116 (23.4)	60 (21.1)	-	-
Hearing aid(s), *n* (%)	Yes	148 (29.8)	137 (59.6)	227 (62.5)	110 (74.8)
Hearing aid use, *n* (%)	No use or < 2 years	-	-	144 (39.7)	39 (26.5)
	≥ 2 years	-	-	219 (60.3)	108 (73.5)
Loneliness, *n* (%)	Moderate to severe	248 (50.1)	134 (47.2)	186 (51.2)	67 (45.6)
Depression, *n* (%)	Moderate to severe	86 (17.3)	45 (15.8)	67 (18.5)	21 (15.6)
Incident falls, *n* (%)	0	433 (87.3)	253 (89.1)	303 (83.5)	120 (81.6)
	1	63 (12.7)	31 (10.9)	60 (16.5)	27 (18.4)
Recurrent falls, *n* (%)	0 or 1	465 (93.8)	263 (92.6)	336 (92.6)	127 (86.4)
	≥ 2	31 (6.3)	21 (7.4)	27 (7.4)	20 (13.6)
Dizziness complaints, *n* (%)	Yes	107 (21.6)	69 (24.3)	96 (26.4)	45 (30.6)
History of dizziness with falls, *n* (%)		55 (11.1)	7 (2.5)	47 (12.9)	6 (4.1)
Chronic health conditions, n (%)	Severe heart disease	29 (5.8)	8 (2.8)	21 (5.8)	4 (2.7)
Hypertension		121 (24.4)	73 (25.7)	89 (24.5)	35 (23.8)
Diabetes		30 (6.0)	12 (4.2)	25 (6.9)	10 (6.8)
Chronic back pain		87 (17.5)	40 (14.1)	75 (20.7)	18 (12.2)
Osteoarthritis of knees and hips		177 (35.7)	72 (25.4)	148 (40.8)	36 (24.5)
Rheumatoid arthritis		37 (7.5)	14 (4.9)	39 (10.7)	2 (1.4)
Other chronic arthritis		29 (5.8)	16 (5.6)	30 (8.3)	7 (4.8)
Other nervous system disorders		10 (2.0)	2 (0.7)	-	-
Malignant condition or cancer		22 (4.4)	17 (6.0)	16 (4.4)	7 (4.8)
Injury due to accident		47 (9.5)	16 (5.6)	41 (11.3)	8 (5.4)
Missing		1 (0.2)	1 (0.3)	0	0
Other health conditions, *n* (%)	Near- or farsightedness	117 (23.6)	51 (18.0)	112 (29.7)	42 (28.6)
Missing		0	0	4	0
Obesity		70 (14.1)	38 (13.4)	66 (18.2)	21 (14.3)
Missing		3 (0.6)	3 (1.0)	0	2 (1.3)
Mobility issues, *n* (%)		113 (22.8)	51 (18.0)	87 (24.0)	25 (17.0)
Difficulty performing usual activities, *n* (%)		126 (25.4)	80 (28.2)	103 (28.4)	40 (27.2)
Missing		1 (0.2)	1 (0.3)	0	0
Alcohol, *n* (%)	> 2 glasses per drinking occasion	199 (40.1)	117 (41.2)	158 (43.5)	67 (46.5)
	Missing	3 (0.6)	9 (3.2)	3 (0.9)	5 (2.5)

All variables were measured at T2 or T3, except baseline SRTn, age and sex, which were measured at T0 and T1. Empty cells (-) represent variables not relevant for that particular research question or too few cases (< 10)

*M* mean, *SD* standard deviation, *N* total sample size, *n* subsample size, *RQ* research question, *ΔSRTn* change in speech reception threshold in noise, *SNR* signal-to-noise ratio, *dB* decibel, T0, baseline measurement round, T1 5-year measurement round, T2 10-year measurement round, T3 15-year measurement round, T0-T2 10-year time interval using data from baseline and the 10-year measurement round, T1-T3 10-year time interval using data from the 5-year and 15-year measurement rounds

In total, *n* = 592 unique participants were included for RQ-1 and 2, with *n* = 188 participants overlapping between the two intervals (see SM Fig. 1). The following statistics are presented for T0-T2 (*n* = 496) and T1-T3 (*n* = 284) samples, respectively. For RQ-1 and 2, mean(SD) baseline SRTn’s were -5.57(2.61) and -5.40(2.66). The ΔSRTn’s over time were 0.62(2.63) and 0.58(2.61). At T2 and T3, 29.8% and 59.6% reported hearing aid use, and 21.6% and 24.3% suffered from dizziness. At T2 and T3, 12.7% and 10.9% reported falling at least once during the past year, and 6.3% and 7.4% reported recurrent falls.

In total *n* = 422 unique participants were included for RQ-3, with *n* = 88 participants overlapping between the two intervals (see SM Fig. 2). The following statistics are presented for T0-T2 (*n* = 363) and T1-T3 (*n* = 147) samples, respectively. At T2 and T3, 39.7% and 26.5% were non-users or used a hearing aid for < 2 years, 60.3% and 73.5% used a hearing aid for ≥ 2 years, 93.9% and 91.8% had an insufficient or poor baseline SRTn score, and 26.4% and 30.6% suffered from dizziness. At T2 and T3, 16.5% and 18.4% reported falling at least once during the past year, and 7.4% and 13.6% reported recurrent falls.

### RQ-1: Association between baseline SRTn and falls 10 years later

In the crude model, baseline SRTn was significantly associated with a 2.27 (95% CI 1.29, 3.99]) and 3.49 (95% CI 1.52, 8.00) higher odds of incident and recurrent falls 10 years later, respectively (see Table 2). Obesity was found to significantly modify the association between baseline SRTn and incident falls 10 years later (Wald Chi-Square[degrees of freedom] = 8.29[3], *p* = 0.040) in the crude model. After stratifying by BMI (< 30 and ≥ 30), among those with a BMI ≥ 30, individuals in the poorest baseline SRTn category (-4.0 to 1.9 dB SNR) had 14.7 (95% CI 2.12, 103) times higher odds for incident falls 10 years later compared to those in the best baseline SRTn category (-10.6 to -7.4 dB SNR).

**Table 2:** Association between baseline SRTn and falls 10 years later (RQ-1)

	Incident falls (1)			Recurrent falls (≥ 2)		
	1 Fall/ 0 falls^e^	OR [95% CI]	*p*	≥ 2 Falls/ < 2 falls^e^	OR [95% CI]	*p*
Model 1						
Baseline SRTn (dB SNR)		-	0.003**^a^		-	0.030*^a^
-10.6 to -7.4 (reference)	22/168	1		6/198	1	
-7.2 to -6.2	20/198	1.00 [0.55, 1.83]	0.99	14/178	2.61 [1.14, 5.94]	0.023*
-6.0 to -4.2	26/170	0.94 [0.50, 1.79]	0.86	12/178	2.05 [0.90, 4.67]	0.087
-4.0 to 1.9	26/150	2.27 [1.29, 3.99]	0.004**	20/174	3.49 [1.52, 8.00]	0.003*
Model 2^b^						
Baseline SRTn (dB SNR)	-	-	-		-	0.12^a^
10.6 to -7.4 (reference)	-	-	-	6/197	1	
-7.2 to -6.2	-	-	-	14/178	2.15 [0.90, 5.13]	0.086
-6.0 to -4.2	-	-	-	12/178	1.79 [0.64, 4.35]	0.20
-4.0 to 1.9	-	-	-	20/174	2.91 [1.21, 7.02]	0.017*
Stratified model						
BMI < 30^c^		-	0.32^a^	-	-	-
Baseline SRTn (dB SNR)						
10.6 to -7.4 (reference)	18/163	1		-	-	-
-7.2 to -6.2	17/149	0.96 [0.50, 1.86]	0.91	-	-	-
-6.0 to -4.2	16/144	0.86 [0.42, 1.75]	0.68	-	-	-
-4.0 to 1.9	25/136	1.50 [0.80, 2.80]	0.21	-	-	-
BMI ≥ 30^d^		-	0.002**^a^	-	-	-
Baseline SRTn (dB SNR)						
10.6 to -7.4 (reference)	1/21	1		-	-	-
-7.2 to -6.2	1/25	0.89 [0.07, 12.1]	0.93	-	-	-
-6.0 to -4.2	3/25	2.39 [0.27, 20.9]	0.43	-	-	-
-4.0 to 1.9	13/19	14.7 [2.12, 103]	0.007**	-	-	-

Model 1 represents the unadjusted model and Model 2 adjusted for confounders

*OR* odds ratio, *CI* confidence interval, *SRTn* speech reception threshold in noise, *dB* decibel, *SNR* signal-to-noise ratio, *BMI* body mass index, *RQ* research question, *ΔSRTn* change in speech reception threshold in noise

^a^Represents *p*-values for Type III fixed effect for baseline SRTn

^b^Adjusted for dizziness and injury

^c^Adjusted for chronic back pain

^d^Adjusted for osteoarthritis of the knees/hips/hands

^e^Represents number of participants who reported falls per baseline SRTn quartile

^*^p < 0.05

^**^p < 0.01

After adjusting for confounders, the poorest baseline SRTn category was significantly associated with recurrent falls 10 years later (OR = 2.91 [95% CI 1.21, 7.02]), compared to the best baseline SRTn category (-10.6 to -7.4 dB SNR).

### RQ-2a: Association between 10-year change in SRTn and falls

No statistically significant association was found between ΔSRTn and incident falls in the crude model (see Table 3). After adjusting for confounders, the odds of recurrent falls were 2.20 [95% CI 1.03, 4.71] times higher among participants with a ΔSRTn between 2.2 and 8.6 dB (worsening hearing) compared to those with a ΔSRTn between -8.8 and -1.0 dB (improved hearing).

**Table 3:** Association between 10-year change in SRTn and falls (RQ-2a)

	Incident falls (1)			Recurrent falls (≥ 2)		
	1 Fall/ 0 falls ^b^	OR [95% CI]	*p*	≥ 2 Falls/ < 2 falls^b^	OR [95% CI]	*p*
Model 1						
ΔSRTn (dB SNR)		-	0.32^a^		-	0.28^a^
-8.8 to -1.0 (reference)	22/168	1		10/180	1	
-1.0 to 0.4	20/198	0.77 [0.41, 1.46]	0.43	11/207	0.89 [0.38, 2.10]	0.80
0.4 to 2.2	26/170	1.09 [0.60, 1.98]	0.77	16/180	1.35 [0.65, 2.80]	0.42
2.2 to 8.6	26/150	1.36 [0.75, 2.46]	0.31	15/161	1.70 [0.84, 3.42]	0.14
Model 2						
ΔSRTn (dB SNR)		-	0.49^a^		-	0.10^a^
-8.8 to -1.0 (reference)	22/167	1		10/180	1	
-1.0 to 0.4	20/197	1.04 [0.51, 2.11]	0.52	11/207	1.17 [0.45, 3.05]	0.75
0.4 to 2.2	26/170	1.33 [0.68, 2.59]	0.40	16/180	1.94 [0.85, 4.46]	0.12
2.2 to 8.6	26/150	1.49 [0.80, 2.79]	0.21	15/161	2.20 [1.03, 4.71]	0.042*

Model 1 represents the unadjusted model and Model 2 adjusted for confounders. Model 2 for incident falls is adjusted for baseline SRTn, hearing aid use, and problems with daily activities. Model 2 for recurrent falls is adjusted for baseline SRTn

*OR* odds ratio, *CI* confidence interval, *ΔSRTn* change in speech reception threshold in noise, *dB* decibel, *SNR* signal-to-noise ratio, *RQ* research question

^a^Represents *p*-values for Type III fixed effect for ΔSRTn

^b^Represents number of participants who reported falls per ΔSRTn quartile

^*^p < 0.05

### RQ-2b: Role of dizziness in the association between 10-year change in SRTn and falls

The interaction between ΔSRTn and dizziness was tested to identify the presence of effect modification. Results showed that the association between ΔSRTn and both incident (Wald Chi-Square[degrees of freedom] = 0.9[3], *p* = 0.804) and recurrent falls (5.25 [3], *p* = 0.154) did not significantly differ between individuals with and without dizziness.

### RQ-3: Association between hearing aid use and falls

In the crude model, the odds of incident falls among individuals with ≥ 2 years hearing aid use did not significantly differ compared to those with < 2 years of use or no use (Table 4). No significant effect modifiers nor confounders were identified for the association between hearing aid use and incident falls.

**Table 4:** Cross-sectional association between hearing aid use and falls after 10 years (RQ-3)

		Incident falls (1)				Recurrent falls (≥ 2)	
			OR [95% CI]	*p*		OR [95% CI]	*p*
Model 1		1 Fall/ 0 falls^a^			≥ 2 Falls/ < 2 falls^a^		
Hearing aid use	No use or < 2 years (reference)	27/156	1		15/168	1	
	≥ 2 years	60/267	1.20 [0.71, 2.01]	0.50	32/295	0.86 [0.39, 1.89]	0.71
Stratified model							
Male							
Hearing aid use	No use or < 2 years (reference)	-	-	-	8/60	1	
	≥ 2 years	-	-	-	9/123	0.46 [0.14, 1.55]	0.21
Female							
Hearing aid use	No use or < 2 years (reference)	-	-	-	7/108	1	
	≥ 2 years	-	-	-	23/172	1.67 [0.94, 2.98]	0.082

Model 1 represents the unadjusted model

*OR* odds ratio, *CI* confidence interval, *RQ* research question

^a^Represents number of participants who reported falls per hearing aid use category

^*^p < 0.05

In the crude model, the odds of recurrent falls among individuals with ≥ 2 years of hearing aid use did also not significantly differ compared to those with < 2 years of use or no use. However, because the interaction between hearing aid use and sex was statistically significant for recurrent falls (Wald Chi-Square[degrees of freedom] = 4.22[3], *p* = 0.040), analyses stratified by sex were performed (Table 4). The odds of recurrent falls between participants with ≥ 2 years of hearing aid use and those with < 2 years or no use did not significantly differ in neither males nor females.

### Sensitivity analyses

GEE results and interpretations of the sensitivity analyses can be found in SM Tables 1 and 4 . No medication use (nonusers) was used as the reference group. Medication users (vs. no users) had a higher odds of incident (OR:1.67, 95% CI 0.93, 3.02) but not recurrent falls (SM Table 3). Out of the types of medication use, only fall risk-related medications were significantly associated with incident (OR:2.08 95% CI 1.01, 4.28) but not recurrent falls. Fall risk-related medication use (OR:2.14, 95% CI 1.08, 4.21) was also associated with worsening of SRTn (ΔSRTn of 2.2 to 8.6 dB SNR) (SM Table 4). However, due to missing T3 data on medication use, further assessment of medication use in the main analyses was considered not possible (see SM for additional explanation).

## Discussion

### Interpretation of findings

#### RQ-1 Baseline SRTn and falls after 10 years

When assessing the association between baseline SRTn and falls 10 years later, we found that individuals with poor hearing at baseline had a significantly higher odds of falling 10 years later, compared to those with good hearing at baseline. This was in agreement with prior studies that have found a significant relationship between hearing impairment at baseline and suffering from falls after one [57], five [35], and seven [58] years. This association also differed by obesity status. An increased odds of incident falls among obese individuals with poor baseline SRTn in our study may signify that a combination of muscle weakness often associated with obesity [59] and a compromised vestibular system caused by hearing impairment results in a higher fall risk. To our knowledge, no prior studies have compared this association between obese and non-obese individuals. More research is needed to confirm this result. Recurrent falls were also significantly associated with baseline SRTn. After adjustment for dizziness and injury, the association with the poor SRTn category remained, showing that adults with severe hearing impairment have an increased risk for recurrent falls 10 years later, independent of other factors.

#### RQ-2a Change in SRTn and falls after 10 years and RQ-2b (Role of dizziness on this association)

When assessing the association between ΔSRTn and falls after 10 years, both unadjusted and adjusted analyses revealed that a 10-year ΔSRTn was not significantly associated with an increased odds of incident falls, compared to no falls. However, adjusted analyses did reveal that those with worsening hearing over 10 years (between 2.2 – 8.6 dB SNR) had significantly higher odds of recurrent falls (i.e., ≥ 2 during the past year). Possible explanations for this association are that worsening hearing over time leads to loss of auditory perception and cues resulting in reduced spatial awareness [19], a reduced cognitive capacity for balance [16–18], and an increased postural sway reflective of hearing loss during the aging process. [60] These factors may more persistently cause vestibular problems and recurrent falls, rather than incidental falls caused by factors unrelated to inner ear dysfunction. Additional research is needed to confirm these explanations.

Earlier studies within the NL-SH [15] and others [10, 61] have found that dizziness was associated with falls. To understand whether dizziness interacts with a change of SRTn on the odds of falling, RQ-2b examined this association between those with and without self-reported dizziness complaints. Results showed that the relationship between change in hearing and falls did not differ between those with and without dizziness complaints. The combination of worsening of hearing ability with symptoms of dizziness therefore did not seem to increase the odds of falling. Perhaps some individuals with both a deterioration in hearing ability and dizziness are more aware of their vestibular problems that could lead to falls, and are therefore more cautious. Moreover, our study had too few cases to further examine whether self-reported types of hearing impairment and frequency of dizziness had any impact on the relationship between hearing ability and falls. Studies with detailed information on the origin and types of dizziness is needed to clarify the relationship between hearing ability and risk of falls.

#### RQ-3 Hearing aid use and falls after 10 years

Among individuals who used a hearing aid for ≥ 2 years, the odds ratio for incident falls was 1.20, whereas for recurrent falls, the odds ratio was 0.86. Although these results were not statistically significant, they are comparable with those of Riska et al. with the slight difference that they assessed daily hearing aid use during the past year [37]. Other studies reported mixed results, showing that hearing aid use (vs. no use) either reduced fall diagnosis/risk [32–34], increased it [35, 36], or had no statistically significant effect [37]. For example, Gopinath et al. found that HA-users at baseline (vs. no users) had an increased risk of ≥ 2 falls 5 years later after adjusting for sex and age, although information about the severity of hearing loss among hearing aid participants was not reported which could potentially explain these results [35]. Assuming that Gopinath’s participants used hearing aids for at least 5 years, perhaps our study’s chosen interval of ≥ 2 years of hearing aid use did not result in significant differences in fall risk when comparing to the reference group. Perhaps it takes more time to detect a beneficial effect of a hearing aid on preventing falls. Studies that assessed the effect of hearing aid use (for at least 3 months) on balance also found mixed results [33, 36]. Perhaps vestibular problems develop independently alongside hearing loss, on which hearing aid use would have little effect. More research on the temporal relationship between balance and hearing loss as well as hearing aid use and its impact on falls due to balance problems is needed.

In the present study, sex significantly modified the association between hearing aid use and recurrent falls, but after performing stratified analyses, no significant associations were found among males nor females. These results are contrary to Lopez et al. who, although did not examine hearing aid use directly, found that hearing impairment was associated with an increased fall risk among both men and women [62]. Our results may be due to a number of factors, such as different hearing aid adjustment periods (adapting to auditory inputs) between men and women. Men and women may differ in whether they withdraw from social/physical activities due to hearing difficulties, potentially affecting mobility levels [63]. After stratifying by sex, the number of participants with ≥ 2 falls during the past year was also very small relative to the number of people who reported < 2 falls. This affected our ability to detect a significant effect. While hearing aid use was not found to be significantly associated to incident nor recurrent falls, the contrasting odds ratios found between the male and female participant groups are noteworthy and warrant further investigation.

### Strengths and limitations

The present study has various strengths. While many studies have investigated the cross-sectional association between hearing ability and falls, to our knowledge this is the first study to examine the relationship between 10-year ΔSRTn and risk of falls. In prior cross-sectional studies, hearing impairment and falls were measured simultaneously using pure-tone audiometry [3, 64, 65], self-reported hearing loss [37, 62, 66–68], and clinical records [69]. Contrarily, our study has the advantage that its longitudinal design allows us to build evidence towards a causal relationship between change in hearing and falls. Additionally, three prior studies have used one [57], five [35], and seven [58] year timeframes to assess hearing impairment at baseline and falls, while our study’s 10-year timeframe has allowed us to assess this relationship over a longer period of time. Given that the average decline in SRTn over 10 years is approximately 0.9 dB SNR [26], this gave us a reliable timeframe to assess its impact on falls. Moreover, contrary to self-reported hearing or pure-tone audiometry, this is the first study to use ΔSRTn to assess its longitudinal relationship with falls, adding to the novelty of the study and our results confirm the usability of SRTn in identifying individuals at risk of falls.

This study has limitations. Firstly, participants were only asked whether they experienced falls due to balance problems during the past year, which limited our ability to assess all-cause falls. Also, information on falls was self-reported, thus, subject to measurement error due to recall bias. Future studies measuring monthly falls, yearly SRTn across a 10-year period, and data on other medical factors that are related to both hearing loss and fall risk (such as reduced cognition and comorbidities) could enhance the reliability of the data necessary to assess temporality and approximate a causal relationship more closely. When interpreting causality, it is also important to consider that a worsening of SRTn over time may itself not cause falls but could reflect a deterioration of the cochlea and the vestibular system together as one anatomical unit. A better measure of this deterioration would represent the relationship between hearing and falls better. Dizziness being an umbrella term, is also non-specific and can indicate various symptoms. This means that those who reported experiencing dizziness may form a heterogeneous group. It is a possibility that some participants may have reported having dizziness symptoms but actually had balance issues or vertigo in mind when answering the survey question, rather than dizziness as a distinct symptom as defined by Bisdorff et al. [24] (pp7). This implies that there may be some overlap of these symptoms which may result in misclassification of dizziness for some participants during statistical analyses for RQ-2. If an interaction effect between change in SRTn and dizziness on the association between change in SRTn and falls exists, and this interaction leads to increased odds of falls among participants who experience dizziness, misclassification of dizziness would attenuate the regression coefficient of this interaction. This may have therefore contributed to insignificant findings for RQ-2. Future studies would benefit from obtaining more precise, distinct measurements of vestibular symptoms and collecting additional details on the timing and triggers of dizziness [70]. Moreover, other unmeasured lifestyle factors such as the home setting [71] and social engagement [72] may also add valuable context to assessing fall risk in future studies.

Additionally, the maximum score of the hearing test is set at + 4 dB SNR. We excluded participants with a baseline SRTn ≥ 2 dB, to leave room for measurable decline, but may have missed declines that would put SRTn beyond + 4 dB. Nevertheless, we believe the range of change in hearing ability over time is sufficiently large to make a reliable estimation of the true association between hearing ability and falls. A quarter of participants showed an improvement in hearing over 10 years, which was used as our reference category. Although an improvement in hearing is possible, such as after removal of excess earwax or surgery, it is unlikely this was the case for all participants in this quartile. Similarly, a worsening in SRTn of up to 8.6 dB SNR as was seen in the highest quartile is much more than average. Despite multiple validations of the National Hearing Test under controlled conditions [73, 74], additional measurement error due to variations within the home setting may have affected the validity of SRTn measured. However, while SRTn may not reflect all parts of the auditory system, it is a clinically relevant and valid indicator of hearing ability and has been shown in prior research to correlate strongly with pure tone average results [40] and better predict self-reported hearing ability compared to pure-tone audiometry [55].

Furthermore, we were unable to assess the impact of medication use at the time of falls as this data was only available at T2 but not T3. It was deemed unfeasible to apply multiple imputation methods for missing T3 data as we had no information on whether medication use changed over 5 years and whether changes in prescription guidelines affected medication dissemination. Moreover, participants were asked to list medications they used during the past *4 weeks*, which created inconsistency when analyzing its association with falls during the *past year*. Lastly, we did not have enough data to examine specificities of hearing aid use, such as fitting, satisfaction, consistency of use, and replacement of hearing aids.

## Conclusion

Individuals with poor SRTn at baseline had a higher odds of incident and recurrent falling 10 years later. Importantly, the co-occurrence of obesity and having poor SRTn at baseline was a significant predictor of increased incident falls 10 years later. Moreover, individuals with a substantial worsening in SRTn over 10 years had a higher odds of recurrent falls. Further prospective research that collects additional details on causes of dizziness could provide a better insight into the role of vestibular symptoms on the relationship between hearing impairment and falls. Additional studies looking into the gender or sex-specific differences in hearing aid use patterns [75] on fall risk as well as the co-occurrence of obesity and hearing impairment on fall risk would benefit fall-risk prevention programs. However, considering the potential risks and gains of addressing hearing impairment in fall prevention programs, such a recommendation seems reasonable based on the available evidence. With the high prevalence of older adults with hearing impairment as well as fall-related injuries, raising awareness among health professionals of the importance of worsening hearing as a risk factor for falls due to balance problems is imperative.

### Supplementary Information

**Supplementary Material 1.**

## Data Availability

The datasets generated and/or analyzed during the current study are not publicly available but are available from the corresponding author upon reasonable request.

## Abbreviations

4DSQ: (Four Dimensional Symptom Questionnaire)
T1: 5-Year measurement round
T2: 10-Year measurement round
T3: 15-Year measurement round
T0: Baseline
BMI: Body mass index
CI: Confidence interval
dB: Decibels
GEE: Generalized Estimating Equations
HL: Hearing loss
kHz: Kilohertz
M: Mean
NL-SH: Netherlands Longitudinal Study on Hearing
OR: Odds ratio
PTA: Pure tone average
RQ: Research Question
SNR: Signal-to-noise ratio
SD: Standard deviation
SRTn: Speech reception threshold-in-noise
SM: Supplementary Material

## References

[1] Konrad HR, Girardi M, Helfert R (1999). Balance and aging. Laryngoscope.

[2] Howarth A, Shone GR (2006). Ageing and the auditory system. Postgrad Med J.

[3] Girard SA, Leroux T, Verreault R, Courteau M, Picard M, Turcotte F (2014). Falls risk and hospitalization among retired workers with occupational noise-induced hearing loss. Can J Aging [Internet].

[4] Cuevas-Trisan R (2017). Balance problems and fall risks in the elderly. Phys Med Rehabil Clin N Am [Internet].

[5] Karlsson MK, Magnusson H, von Schewelov T, Rosengren BE (2013). Prevention of falls in the elderly–a review. Osteoporos Int [Internet].

[6] VeiligheidNL. Feiten en cijfers valongevallen 65-plussers 2020 . Veiligheid.nl. 2020. Available from: https://www.veiligheid.nl/sites/default/files/2022-04/Infographic%20cijfers%20valongevallen%202020_0.pdf Accessed 26 June 2023.

[7] Peeters G, van Schoor NM, Cooper R, Tooth L, Kenny RA (2018). Should prevention of falls start earlier? Co-ordinated analyses of harmonised data on falls in middle-aged adults across four population-based cohort studies. PLoS One [Internet].

[8] Stam C. Letsels 2021 [Internet]. 2022. Available from: https://www.veiligheid.nl/sites/default/files/2023-02/Kerncijfers%20Letsels%202021%20versie%202.pdf Accessed 26 June 2023.

[9] Chang VC, Do MT (2015). Risk factors for falls among seniors: implications of gender. Am J Epidemiol [Internet].

[10] Alyono JC (2018). Vertigo and dizziness. Otolaryngol Clin North Am.

[11] Petersen N, König H-H, Hajek A (2020). The link between falls, social isolation and loneliness: A systematic review. Arch Gerontol Geriatr [Internet].

[12] Carpenter MG, Campos JL. The effects of hearing loss on balance: A critical review. Ear Hear . 2020;41 Suppl 1(Supplement 1):107S-119S. doi:10.1097/AUD.0000000000000929 10.1097/AUD.0000000000000929 33105265

[13] Foster JI, Williams KL, Timmer BHB, Brauer SG (2022). The association between hearing impairment and postural stability in older adults: A systematic review and meta-analysis. Trends Hear [Internet].

[14] Heitz ER, Gianattasio KZ, Prather C, Talegawkar SA, Power MC (2019). Self-reported hearing loss and nonfatal fall-related injury in a nationally representative sample: Self-reported hearing loss and fall-related injury. J Am Geriatr Soc [Internet].

[15] Stam M, Kostense PJ, Lemke U, Merkus P, Smit JH, Festen JM (2014). Comorbidity in adults with hearing difficulties: which chronic medical conditions are related to hearing impairment?. Int J Audiol [Internet].

[16] Koh DH, Lee JD, Lee HJ (2015). Relationships among hearing loss, cognition and balance ability in community-dwelling older adults. J Phys Ther Sci [Internet].

[17] Mosnier I, Bebear J-P, Marx M, Fraysse B, Truy E, Lina-Granade G (2015). Improvement of cognitive function after cochlear implantation in elderly patients. JAMA Otolaryngol Head Neck Surg [Internet].

[18] Semenov YR, Bigelow RT, Xue Q-L, du Lac S, Agrawal Y. Association between vestibular and cognitive function in U.S. adults: Data from the National Health and nutrition examination survey. J Gerontol A Biol Sci Med Sci . 2016;71(2):243–50. doi:10.1093/gerona/glv069 10.1093/gerona/glv069 PMC5864155 26219850

[19] Keller BK, Morton JL, Thomas VS, Potter JF (1999). The effect of visual and hearing impairments on functional status. J Am Geriatr Soc [Internet].

[20] Jiam NT-L, Li C, Agrawal Y (2016). Hearing loss and falls: A systematic review and meta-analysis: Systematic Review on Hearing Loss and Falls. Laryngoscope.

[21] Davis A, Smith P, Ferguson M, Stephens D, Gianopoulos I (2007). Acceptability, benefit and costs of early screening for hearing disability: a study of potential screening tests and models. Health Technol Assess.

[22] Huang RJ, Pieper CF, Whitson HE, Garrison DB, Pavon JM, Riska KM (2022). Evaluating the association between hearing loss and falls in adults with vestibular dysfunction or nonvestibular dizziness. Ear Hear [Internet].

[23] van de Berg R, Murdin L, Whitney SL, Holmberg J, Bisdorff A (2022). Curriculum for Vestibular Medicine (VestMed) proposed by the Bárány Society. J Vestib Res.

[24] Bisdorff A, Von Brevern M, Lempert T, Newman-Toker DE (2009). Classification of vestibular symptoms: towards an international classification of vestibular disorders. J Vestib Res.

[25] Haile LM, Kamenov K, Briant PS, Orji AU, Steinmetz JD, Abdoli A (2021). Hearing loss prevalence and years lived with disability, 1990–2019: findings from the Global Burden of Disease Study 2019. Lancet [Internet].

[26] Goderie TPM, Stam M, Lissenberg-Witte BI, Merkus P, Lemke U, Smits C (2020). 10-year follow-up results of the Netherlands Longitudinal Study on hearing: Trends of longitudinal change in speech recognition in noise. Ear Hear [Internet].

[27] van der Meijden Martien Panneman Saskia Kloet Birgitte Blatter Nienke Homans André Goedegebure W. Prevalentie van gehoorverlies in Nederland [Internet]. 2020. Available from: https://www.veiligheid.nl/kennisaanbod/onderzoek/rapport-12-miljoen-mensen-leden-2020-aan-gehoorverlies-nederland Accessed 26 June 2023.

[28] Kim SY, Min C, Kim H-J, Lee CH, Sim S, Park B (2020). Mortality and cause of death in hearing loss participants: A longitudinal follow-up study using a National Sample Cohort. Otol Neurotol [Internet].

[29] Tricco AC, Thomas SM, Veroniki AA, Hamid JS, Cogo E, Strifler L (2017). Comparisons of interventions for preventing falls in older adults: A systematic review and meta-analysis. JAMA [Internet].

[30] Mahafza MT, Wilson WJ, Brauer S, Timmer BHB, Hickson L (2022). A systematic review of the effect of hearing aids on static and dynamic balance in adults with Hearing Impairment. Trends Hear [Internet].

[31] Borsetto D, Corazzi V, Franchella S (2021). The Influence of Hearing Aids on Balance Control: A Systematic Review. Audiol Neurootol.

[32] Mahmoudi E, Basu T, Langa K, McKee MM, Zazove P, Alexander N (2019). Can hearing aids delay time to diagnosis of dementia, depression, or falls in older adults?. J Am Geriatr Soc [Internet].

[33] Rumalla K, Karim AM, Hullar TE (2015). The effect of hearing aids on postural stability: Hearing Aids and Balance. Laryngoscope [Internet].

[34] Stevens MN, Barbour DL, Gronski MP, Hullar TE (2016). Auditory contributions to maintaining balance. J Vestib Res [Internet].

[35] Gopinath B, McMahon CM, Burlutsky G, Mitchell P (2016). Hearing and vision impairment and the 5-year incidence of falls in older adults. Age Ageing [Internet].

[36] Weaver TS, Shayman CS, Hullar TE (2017). The effect of hearing aids and cochlear implants on balance during gait. Otol Neurotol [Internet].

[37] Riska KM, Peskoe SB, Gordee A, Kuchibhatla M, Smith SL (2021). Preliminary evidence on the impact of hearing aid use on falls risk in individuals with self-reported hearing loss. Am J Audiol [Internet].

[38] van Wier MF, Jansen LA, Goderie T, Stam M, Nachtegaal J, van Beek JHM (2023). Cohort profile: Netherlands Longitudinal Study on Hearing (NL-SH). BMJ Open [Internet].

[39] Jehu DA, Davis JC, Falck RS, Bennett KJ, Tai D, Souza MF (2021). Risk factors for recurrent falls in older adults: A systematic review with meta-analysis. Maturitas [Internet].

[40] Smits C, Kapteyn TS, Houtgast T (2004). Development and validation of an automatic speech-in-noise screening test by telephone. Int J Audiol [Internet].

[41] Terluin B, van Marwijk HWJ, Adèr HJ, de Vet HCW, Penninx BWJH, Hermens MLM (2006). The Four-Dimensional Symptom Questionnaire (4DSQ): a validation study of a multidimensional self-report questionnaire to assess distress, depression, anxiety and somatization. BMC Psychiatry [Internet].

[42] Gierveld JDJ, Van Tilburg T (2006). A 6-item scale for overall, emotional, and social loneliness: Confirmatory tests on survey data. Res Aging [Internet].

[43] Balestroni G, Bertolotti G (2012). EuroQol-5D (EQ-5D): an instrument for measuring quality of life. Monaldi Arch Chest Dis [Internet].

[44] Nachtegaal J, Smit JH, Smits C, Bezemer PD, van Beek JHM, Festen JM (2009). The association between hearing status and psychosocial health before the age of 70 years: results from an internet-based national survey on hearing. Ear Hear [Internet].

[45] Agmon M, Lavie L, Doumas M (2017). The association between hearing loss, postural control, and mobility in older adults: A systematic review. J Am Acad Audiol.

[46] Chen X, He L, Shi K, Yang J, Du X, Shi K (2023). Age-stratified modifiable fall risk factors in Chinese community-dwelling older adults. Arch Gerontol Geriatr [Internet].

[47] Kvelde T, McVeigh C, Toson B, Greenaway M, Lord SR, Delbaere K (2013). Depressive symptomatology as a risk factor for falls in older people: systematic review and meta-analysis. J Am Geriatr Soc [Internet].

[48] Harvard Health. Medications that increase your risk of falling . Cambridge (USA)(: Harvard Health; 2021. Available from: https://www.health.harvard.edu/staying-healthy/medications-that-increase-your-risk-of-falling Accessed 26 June 2023.

[49] Scott D, Blizzard L, Fell J, Jones G (2009). Statin therapy, muscle function and falls risk in community-dwelling older adults. QJM [Internet].

[50] Seppala LJ, van de Glind EMM, Daams JG, Ploegmakers KJ, de Vries M, Wermelink AMAT (2018). Fall-risk-increasing drugs: A systematic review and meta-analysis: III. Others. J Am Med Dir Assoc.

[51] Seppala LJ, Wermelink AMAT, de Vries M, Ploegmakers KJ, van de Glind EMM, Daams JG, et al. Fall-risk-increasing drugs: A systematic review and meta-analysis: II. Psychotropics. J Am Med Dir Assoc. 2018;19(4):371.e11-371.e17. 10.1016/j.jamda.2017.12.098 . 10.1016/j.jamda.2017.12.098 29402652

[52] de Vries M, Seppala LJ, Daams JG, van de Glind EMM, Masud T, van der Velde N, et al. Fall-risk-increasing drugs: A systematic review and meta-analysis: I. cardiovascular drugs. J Am Med Dir Assoc . 2018;19(4):371.e1–371.e9. doi:10.1016/j.jamda.2017.12.013 10.1016/j.jamda.2017.12.013 29396189

[53] Woolcott JC, Richardson KJ, Wiens MO, Patel B, Marin J, Khan KM (2009). Meta-analysis of the impact of 9 medication classes on falls in elderly persons. Arch Intern Med [Internet].

[54] van Leeuwen LM, Goderie TPM, van Wier MF, Lissenberg-Witte BI, Lemke U, Kramer SE (2021). Uptake of hearing aids and hearing assistive technology in a working population: Longitudinal analyses of the Netherlands Longitudinal Study on hearing. Ear Hear [Internet].

[55] Smits C, Kramer SE, Houtgast T (2006). Speech reception thresholds in noise and self-reported hearing disability in a general adult population. Ear Hear [Internet].

[56] ter Wee MM, Lissenberg-Witte BI (2019). A quick guide on how to conduct medical research: From set-up to publication.

[57] Kulmala J, Viljanen A, Sipilä S, Pajala S, Pärssinen O, Kauppinen M (2009). Poor vision accompanied with other sensory impairments as a predictor of falls in older women. Age Ageing [Internet].

[58] Purchase-Helzner EL, Cauley JA, Faulkner KA, Pratt S, Zmuda JM, Talbott EO (2004). Hearing sensitivity and the risk of incident falls and fracture in older women: the study of osteoporotic fractures. Ann Epidemiol [Internet].

[59] Tinetti ME, Inouye SK, Gill TM, Doucette JT (1995). Shared risk factors for falls, incontinence, and functional dependence. Unifying the approach to geriatric syndromes. JAMA.

[60] Gandemer L, Parseihian G, Kronland-Martinet R, Bourdin C (2017). Spatial cues provided by sound improve postural stabilization: Evidence of a spatial auditory map?. Front Neurosci [Internet].

[61] Tuunainen E, Rasku J, Jäntti P, Pyykkö I (2014). Risk factors of falls in community dwelling active elderly. Auris Nasus Larynx [Internet].

[62] Lopez D, McCaul KA, Hankey GJ, Norman PE, Almeida OP, Dobson AJ (2011). Falls, injuries from falls, health related quality of life and mortality in older adults with vision and hearing impairment–is there a gender difference?. Maturitas [Internet].

[63] Viljanen A, Kaprio J, Pyykkö I, Sorri M, Koskenvuo M, Rantanen T (2009). Hearing acuity as a predictor of walking difficulties in older women. J Am Geriatr Soc.

[64] Assantachai P, Praditsuwan R, Chatthanawaree W, Pisalsarakij D, Thamlikitkul V (2003). Risk factors for falls in the Thai elderly in an urban community. J Med Assoc Thai.

[65] Lin FR, Ferrucci L (2012). Hearing loss and falls among older adults in the United States. Arch Intern Med [Internet].

[66] Riska KM, Peskoe SB, Kuchibhatla M, Gordee A, Pavon JM, Kim SE (2022). Impact of hearing aid use on falls and falls-related injury: Results from the health and Retirement Study: Results from the health and retirement study. Ear Hear [Internet].

[67] Zhou Y, Hu Y, Luo J, Li Y, Liu H, Sun X, et al. Association between sensory loss and falls among middle-aged and older Chinese population: Cross-sectional and longitudinal analyses. Front Med (Lausanne). 2021;8:810159. 10.3389/fmed.2021.810159 PMC8793905 35096898

[68] Sihvonen S, Era P, Helenius M (2004). Postural balance and health-related factors in middle-aged and older women with injurious falls and non-fallers. Aging Clin Exp Res [Internet].

[69] Bumin G, Uyanik M, Aki E, Kayihan H (2002). An investigation of risk factors for fails in elderly people in a Turkish rest home: a pilot study. Aging Clin Exp Res [Internet].

[70] Newman-Toker D. E, Edlow J. A (2015). TiTrATE: A Novel, Evidence-Based Approach to Diagnosing Acute Dizziness and Vertigo. Neurologic clinics.

[71] Campani D, Caristia S, Amariglio A, Piscone S, Ferrara LI, Barisone M (2021). Home and environmental hazards modification for fall prevention among the elderly. Public Health Nurs [Internet].

[72] Holman JA, Drummond A, Naylor G (2021). Hearing aids reduce daily-life fatigue and increase social activity: A longitudinal study. Trends Hear [Internet].

[73] Folmer RL, Vachhani J, McMillan GP, Watson C, Kidd GR, Feeney MP (2017). Validation of a computer-administered version of the digits-in-noise test for hearing screening in the United States. J Am Acad Audiol [Internet].

[74] Smits C, Merkus P, Houtgast T (2006). How we do it: The Dutch functional hearing-screening tests by telephone and internet. Clin Otolaryngol [Internet].

[75] Reavis KM, Bisgaard N, Canlon B (2023). Sex-Linked Biology and Gender-Related Research Is Essential to Advancing Hearing Health. Ear Hear.

