# Supplementary material for: Ten-year association between change in speech-in-noise recognition and falls due to balance problems: a longitudinal cohort study
Source: BMC Public Health. 2024 Mar 7;24:732. doi: 10.1186/s12889-024-18187-5 (PMC10919036; doi:10.1186/s12889-024-18187-5)
Supplement: Supplementary file 1 — Supplementary Material 1. [file 12889_2024_18187_MOESM1_ESM.docx]

**Supplementary Material to:**

Ten-Year Association between Change in Speech-in-Noise Recognition and Falls due to Balance Problems: A Longitudinal Cohort Study

Lotte A. Jansen*^1,2^, Marieke F. van Wier*^1,2^, Freek P.J. Vernimmen^1,2^,

Thadé Goderie^1,2^, Raymond van de Berg^4^, Ulrike Lemke^5^,

Birgit I. Lissenberg-Witte^3^, Sophia E. Kramer^1,2^

^1^Amsterdam UMC location Vrije Universiteit Amsterdam, Department of Otolaryngology – Head and Neck Surgery, section Ear and Hearing, De Boelelaan 1117, Amsterdam, the Netherlands

^2^Amsterdam Public Health Research Institute, Quality of Care, Amsterdam, the Netherlands

^3^Amsterdam UMC location Vrije Universiteit Amsterdam, Department of Epidemiology and Data Science, Meibergdreef 9, Amsterdam, the Netherlands

^4^Division of Balance Disorders, Department of Otorhinolaryngology and Head and Neck Surgery, Maastricht University Medical Centre, P. Debyelaan 25, Maastricht, The Netherlands

^5^Research & Development, Sonova AG, Staefa, Switzerland

* These authors contributed equally to this work

**T0-T2**

**T1-T3**

Participants with data available at T0

(prior to January 2023)

N = 3024

Participants with data available at T0

(prior to January 2023)

N = 3024

n = 496

n = 284

n = 504

n = 289

n = 303

n = 347

n = 347

n = 391

n = 448

n = 460

n = 519

n = 544

n = 628

n = 673

n = 885

n = 890

Missing HT data at T1 and/or T3

n = 2564

Missing HT data at T0 and/or T2

n = 2134

Missing fall data at T3

n = 12

Missing fall data at T2

n = 5

< 40 years of age at T0

n = 212

< 40 years of age at T1

n = 57

Cochlear implant use at T0, T1, or T2

n = 45

Cochlear implant use at T0, T1, T2, or T3

n = 44

Performed HT with HA at T1 or T3

n = 0

Performed HT with HA at T0 or T2

n = 84

SRTn ≥ 2 dB SNR at T1

n = 44

SRTn ≥ 2 dB SNR at T0

n = 25

Congenital hearing loss

n = 15

Congenital hearing loss

n = 14

10-year ΔSRTn > 3 IQR

n = 8

n = 25

10-year ΔSRTn > 3 IQR

n = 5

n = 188*

**Fig. 1**. Flowchart of participant numbers for RQ-1 (association baseline SRTn and falls), RQ-2a (association ΔSRTn and falls after 10y), and 2b (role of dizziness).

*Note.* T0-T2 refers to the 10-year time interval using data from baseline and the 10-year measurement round. T1-T3 refers to the 10-year time interval using data from the 5-year and 15-year measurement rounds.

*Represents participants overlapping both time intervals (T0-T2 and T1-T3).

Abbreviations. HT, hearing test; SRTn, speech reception threshold in noise; dB, decibels; SNR, speech in noise recognition; HA, hearing aid; Δ, change; N, total sample size; n, subsample size; IQR, interquartile range; T0, baseline measurement round; T1, 5-year measurement round; T2, 10-year measurement round; T3, 15-year measurement round.

**T0-T2**

**T1-T3**

Participants with data available at T0

(prior to January 2023)

N = 3024

Participants with data available at T0

(prior to January 2023)

N = 3024

n = 277

n = 291

n = 333

n = 380

n = 460

n = 1445

n = 688

n = 704

n = 754

n = 998

n = 1006

n = 2934

Missing HT at T1

n = 1579

No fall data at T3

n = 985

Missing HT at T0

n = 90

n = 363

No fall data at T2

n = 1928

Missing data on duration of HA use

n = 80

Missing data on duration of HA use

n = 8

< 40 years of age at T1

n = 47

< 40 years of age at T0

n = 244

Cochlear implant use at T0, T1, T2, or T3

n = 42

Cochlear implant use at T0, T1, or T2

n = 50

Congenital hearing loss

n = 12

Congenital hearing loss

n = 16

SRTn < -5.5 dB SNR (T1) and

no HA use (T1)

n = 130

SRTn < -5.5 dB SNR (T0) and

no HA use (T0)

n = 325

n = 88*

n = 147

**Fig. 2**. Flowchart of participant numbers for RQ-3 (cross-sectional association between hearing aid use and falls).

*Note.* T0-T2 refers to the 10-year time interval using data from baseline and the 10-year measurement round. T1-T3 refers to the 10-year time interval using data from the 5-year and 15-year measurement rounds. Although this sample was used for a cross-sectional analysis, the same 10-year time interval (T0-T2 and T1-T3) approach seen in Fig. 1, Supplementary Material was taken in order to be able to apply some of the exclusion criteria (e.g. age < 40 years) needed from baseline (T0 or T1) data.

*Represents participants overlapping both time intervals (T0-T2 and T1-T3).

Abbreviations. HT, hearing test; SRTn, speech reception threshold in noise; dB, decibels; SNR speech in noise recognition; HA, hearing aid; N, total sample size; n, subsample size; T0, baseline measurement round; T1, 5-year measurement round; T2, 10-year measurement round; T3, 15-year measurement round.

Table 1. Medication classes and their relation to fall risk.

| **Relation to falls** | **Medication classes** |
| --- | --- |
| Risk-related (increasing risk)^a^ | Antidepressants  Antipsychotics  Benzodiazepines  Opioids  Antiepileptics  Proton-pump-inhibitors  NSAIDS  Analgesics  Diuretics  Alpha blockers  Calcium channel blockers  Angiotensin II receptor blockers  Angiotension converting enzyme inhibitors  Sedatives  Hypnotics  Muscle relaxants  Insulin  First-generation antihistamines  Anticholinergics |
| Mixed evidence^,b^ | Beta-blockers  Statins |
| Unrelated (no established relation found in the literature) | Paracetamol  Antibiotics  Corticosteroids  Anticoagulants  Antiplatelets  Antiparkinson’s medications  Second-generation antihistamines  Stimulants  Vitamins |

*Note.* Medication classes were identified from the lists of medications participants provided in the T2 survey. Based on the current literature, they were placed in groups based on their relation to falls. Medications in the row “Mixed evidence” concerns medications that have been reported to decrease fall risk in one or more studies and to increase fall risk in others. In our analyses these were included into the “fall risk-related (increasing)” category rather than a separate “mixed evidence” category due to too few individuals reporting using these medications.

^a^ Seppala et al. 2018; De Vries et al. 2018; Woolcott et al. 2019; Bloch et al. 2011; Harvard Health

^b^ De Vries et al. 2018

^c^ UNMC, 2023; Scott et al. 2009.

Table 2. Descriptive statistics of medication use.

| **Variables** | **T0-T2 (N=394)** | **T1-T3 (N=221)** |
| --- | --- | --- |
| Medication use during past month, n (%)  Yes  No | 261 (66.2)  133 (33.8) | 145 (65.6)  76 (34.4) |
| When using medication: n (%)  Only fall risk-related (increasing) meds  Only fall risk-unrelated meds*  Mix of fall risk-related and -unrelated meds** | 75 (28.7)  54 (20.7)  132 (50.6) | 40 (27.6)  29 (20.0)  76 (52.4) |
| When using medication n (%)  No polypharmacy (1-3 meds)  Polypharmacy (4-10 meds) | 165 (63.2)  96 (36.8) | 91 (62.8)  54 (37.2) |

*Note*. Sensitivity analyses were performed using data of participants who reported on (non-)use of medication. T0-T2 refers to the 10-year time interval using data from baseline and the 10-year measurement round. T1-T3 refers to the 10-year time interval using data from the 5-year and 15-year measurement rounds. Of the full T0-T2 sample available for the main analyses, 98 participants declined to report on medication use, resulting in N=398 available for the sensitivity analyses. At T3, questions on medication use were not included in the survey. Data of the T1-T3 sample were imputed with medication data reported at T2, assuming this medication was also used at T3. Of the full T1-T3 sample available for the main analyses, 62 participants declined to report on medication use at T2, resulting in N=222 available for the sensitivity analyses.

* Participants who used fall-risk increasing medications (includes statins and beta-blockers from “Mixed evidence” row in Table 1), but no other medications.

** Participants who used a mix of medications related to fall risk and medications unrelated to fall risk.

Table 3. Association between medication use and falls.

|  | **Incident falls (1)** | | **Recurrent falls (≥2)** | |
| --- | --- | --- | --- | --- |
|  | OR [95% CI] | *p* | OR [95% CI] | *p* |
| No medication use (reference) Medication use during the past 4 weeks | 1  1.67 [0.93, 3.02] | .  0.082* | 1  1.24 [0.59, 2.61] | .  0.58 |
| No medication use (reference)  Only fall-risk related (increasing) meds | 1  2.08 [1.01, 4.28] | .  0.046* | 1  1.20 [0.44, 3.28] | .  0.72 |
| No medication use (reference) | 1 | . | 1 | . |
| Only fall risk unrelated meds | 1.37 [0.55, 3.41] | 0.50 | 1.11 [0.36, 3.43] | 0.85 |
| No medication use (reference) | 1 | . | 1 | . |
| Mix of fall-risk related and unrelated meds | 1.60 [0.82, 3.09] | 0.17 | 1.32 [0.57, 3.05] | 0.52 |
| No polypharmacy (reference)  Polypharmacy | 1  1.10 [0.60, 2.04] | .  0.76 | 1  1.19 [0.51, 2.75] | .  0.69 |

**p*<0.1.

Abbreviations. OR, odds ratio; CI, confidence interval.

Table 4. Association between medication use and ΔSRTn.

|  | **Medication use during the past 4 weeks** | **OR [95% CI]** | ***p*** |
| --- | --- | --- | --- |
| **ΔSRTn (dB SNR)** |  |  | 0.29^a^ |
| -8.8 to -1.0 (reference) | No use (reference) | 1 | . |
|  | Use | 1 | . |
| -1.0 to 0.4 | No use (reference) | 1 | . |
|  | Use | 1.02 [0.64, 1.62] | 0.93 |
| 0.4 to 2.2 | No use (reference) | 1 | . |
|  | Use | 0.87 [0.55, 1.39] | 0.56 |
| 2.2 to 8.6 | No use (reference) | 1 | . |
|  | Use | 1.55 [0.93, 2.58] | 0.091* |
|  | **Fall risk related medication use** |  |  |
| **ΔSRTn (dB SNR)** |  |  | 0.082*^a^ |
| -8.8 to -1.0 (reference) | No use (reference) | 1 | . |
|  | Fall risk related (increasing) use | 1 | . |
| -1.0 to 0.4 | No use (reference) | 1 | . |
|  | Fall risk related (increasing) use | 1.02 [0.52, 1.99] | 0.96 |
| 0.4 to 2.2 | No use (reference) | 1 | . |
|  | Fall risk related (increasing) use | 1.15 [0.60, 2.21] | 0.68 |
| 2.2 to 8.6 | No use (reference) | 1 | . |
|  | Fall risk related (increasing) use | 2.14 [1.08, 4.21] | 0.028* |

*Note.* Dependent variable is change in SRTn, independent variables are medication use and fall risk-related medication use.

^a^ Represents *p*-values for Type III fixed effect for baseline SRTn.

**p*< 0.1

Abbreviations. OR, odds ratio; CI, confidence interval; ΔSRTn, change in speech reception threshold in noise; dB, decibels; SNR, speech in noise recognition.

*Explanation and interpretation of the results presented in Tables 3 and 4*

For a variable to be considered a possible confounder, it has to have an association with both the dependent and independent variable. Associations between each medication variable and incident and recurrent falls were therefore tested (Table 3) by performing univariate GEE analyses. The results showed that medication use at all (vs. no use) and fall risk-related medication use (a combination of medication that increases risk for falls with medication that decreases risk for falls but no other medication) had statistically significant associations with increased incident fall risk, compared to no medication use. None of the other medication categories had a significant relation with falls. To test whether medication use at all (vs. no use) and fall risk-related medication use were significantly associated with change in SRT, these associations were tested next and were statistically significant (Table 4). However, due to the fact that participants were not asked any questions on medication use during the T3 survey, imputing this missing data and including these medication variables in the final regression models would have resulted in unreliable results; mainly due to the time-varying nature of medication use and inability to accurately predict use at T3.
